# Potential of Community Volunteers in Flood Early Warning Dissemination: A Case Study of Bangladesh

**DOI:** 10.3390/ijerph182413010

**Published:** 2021-12-09

**Authors:** Murtuza Al-Mueed, Md Rafique Ahasan Chawdhery, Emmanuel Harera, Riyadh A. Alhazmi, Abdulmajeed M. Mobrad, Saqer M. Althunayyan, Ahmed M. Al-Wathinani

**Affiliations:** 1 Ministry of Public Administration, Dhaka 1000, Bangladesh; 2 Humanitarian and Conflict Response Institute, School of Arts, Languages and Cultures, University of Manchester, Manchester M13 9PL, UK; hareraemmy@gmail.com; 3 Department of Agroecology and Crop Production, Faculty of Agrobiology, Food and Natural Resources, Czech University of Life Sciences, Prague, Kamýcká 129, Suchdol, 16500 Prague, Czech Republic; chawdhery@af.czu.cz; 4 Department of Emergency Medical Services, Prince Sultan Bin Abdulaziz College Emergency Medical Services, King Saud University, Riyadh 11451, Saudi Arabia; rialhazmi@ksu.edu.sa (R.A.A.); amobrad@ksu.edu.sa (A.M.M.); ahmalotaibi@ksu.edu.sa (A.M.A.-W.); 5 Department of Accident and Trauma, Prince Sultan Bin Abdulaziz College Emergency Medical Services, King Saud University, Riyadh 11451, Saudi Arabia; salthunayyan@ksu.edu.sa

**Keywords:** flood early warning, flood response, community volunteerism, disaster volunteer group, resilience, governance and planning, disaster management, sustainability

## Abstract

Flood early warning (FEW) is a vital component of disaster risk management and is particularly important for saving lives, developing a sustainable agro-based economy, economic stability, and the overall development of the people of Bangladesh as well as others. This study was conducted in a northern, flood-prone area of Bangladesh to investigate the potential of incorporating volunteers of the community to the Union Councils (UCs) to disseminate FEW alongside the top-down approach. Several studies have found that despite having a sophisticated flood forecasting technology, local communities are not reaping the benefits of it, as the existing dissemination system is inaccessible to most local people. Since risk communication takes place in a social context, this study investigated and thereby proposed that volunteerism, as a form of social capital or communal virtue, can potentially assist the community-based disaster management (CBDM) institutions in enhancing their capacity to reach the maximum population at times of flood risk. Therefore, it was confirmed that the trained volunteers need to be integrated into and endorsed by the national policy. In addition, this study also provides a number of recommendations connecting literature with policy documents of Bangladesh.

## 1. Introduction

**Figure 1:**
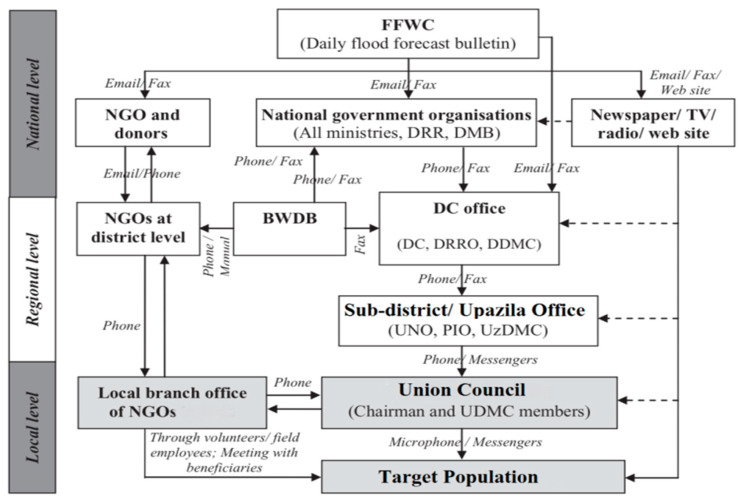
FEW dissemination networks from national to UC in Bangladesh (adopted from Shah et al. [7]).

Flooding is a major natural disaster in Bangladesh, considering the number of people affected as well as the frequency of occurrence. Dissemination of flood risk information is an important part of a flood forecasting and early warning system (FFEWS) [1]. The government of Bangladesh strengthened the Community-Based Disaster Management (CBDM) approach based on the Union Councils (UCs), the lowest tier of local government institutions in the country. Under each UC there is a Union Disaster Management Committee (UDMC) responsible for managing disasters at the community level. However, it was realized that further improvement in the process of disseminating flood information was needed to reach those at immediate flood risk [2].The primary goal of a Flood Early Warning (FEW)system is to increase the safety of the people and reduce the harmful impact of floods [3,4]. People need to know the risk factors, and they should understand the warning to cope with the coming flood [5]. Therefore, a FEW minimizes risk to life, helps to evacuate the danger zone in time, helps to move tangible valuable belongings to a safe place, encourages the taking of flood resilience measures, establishes two-way communication, and provides useful and expert advice. Despite these positive effects, several findings from contemporary studies have shown that individuals and households lack effective flood warnings at the community level in Bangladesh [6]. For an effective flood risk management timely warning, dissemination of flood information and response actions are essential for every community at risk. However, the communities of Bangladesh are not well aware of the FEWSs, and there exists a stark gap between the top-down national flood information flow and the intended recipients of that information. People prefer ‘locally available and easily understandable’ FEW and instructions at the community level of Bangladesh, especially those who are living at the union level (the lowest administrative unit of local government) [6]. Therefore, community participation in planning and implementing decisions of flood risk management needs to be institutionalized, a process which ultimately reduces vulnerability and builds disaster resilience throughout the community. The government of Bangladesh has indeed taken initiatives to institutionalize disaster management at the union level thorough UDMCs. However, the committees largely give importance to the post-flood relief, rescue, and rehabilitation process and less to disseminating FEW. It is not practical for the UDMC to reach all the villages and communities to disseminate a FEW. FEW information flows from the FFWC (Flood Forecasting and Warning Centre) to the union level. Figure 1 shows that FEWs are transferred directly to the target population and to the NGOs who are responsible for local disaster management [7]. It also indicates that NGOs disseminate information through their employees and volunteers to the target population, but no detail is given as to how NGOs and volunteers take part in this process. Considering this lapse, it is necessary to study the role of volunteers in disseminating FEWs in the context of a flood-prone area.

Inevitably people’s participation is required to improve the dissemination process; however, the role of community and neighbors proved to be very effective in a flood-prone area [2]. In connection to a FEW dissemination systems, the findings of a recent study in Azad Jammu and Kashmir, India, revealed that through the formal channels flood warnings did not reach 78 percent of the intended population, a distinct failure to address the needs of the community [8]. Both studies demonstrated that the active participation of the local community is critical to make a dissemination system successful. One of the reasons for failure of a FEW is that while designing the systems the advantages and disadvantages of different channels of FEW dissemination are not taken into account [9]. The most advanced and sophisticated technology does not necessarily lead to optimum outcomes. Furthermore, sufficient education and an uninterrupted electricity supply are important factors to access such technology. Mastor et al.’s [9] study noted the limitations of different channels used for a flood warning dissemination process and identified several disadvantages of technology-based dissemination systems, and these drawbacks very much correspond to the existing socio-economic structure of Bangladesh.

Technology is not a panacea for disaster FEWs [10]. In most developing countries, including Bangladesh, broadcast media, newspapers, and websites are used for the dissemination of flood risk information. However, these channels may not reach the at-risk population in a timely manner and/or provide sufficient information [11]. This technological inaccessibility cannot be solved in the short term, nor it is easy to make everyone aware of the warning system within a short period of time. However, it is possible to bridge the gap in terms of what is technologically available and what is socially available by drawing on social networks and strengthening the bond between official sources and local disaster risk management mechanisms. Volunteerism is a social capital, a common form of instinctive, often unplanned and uncoordinated assistance to help the community [12]. This quality of human behavior is one of the key elements that has significant potential to reduce disaster risk, build resilience, and generate immediate responses to a disaster [13]. Therefore, it is important to include the local community and volunteers when designing flood risk communication [14]. The idea of Community-Based Disaster Management (CBDM) opens a wide scope for individuals to work alongside the dedicated or responsible authorities in the disaster preparedness phase as well as in the response phase. In CBDM, volunteers can be widely incorporated into various activities. For example, in Sri Lanka, community volunteers are trained in hazard mapping, first aid, and building awareness, including trained 1000 volunteers from a community of 4,00,000 people to perform in an early warning system [15]. In Bangladesh, volunteers are also utilized in coastal areas for cyclone early warnings. A project named the Cyclone Preparedness Program trained 32,000 volunteers to disseminate cyclone early warnings [16]. Disaster preparedness involves understanding the nature of the hazard, planning, coordination, training, and leadership at every community, local, state, and national level of an existing decentralized government system, whereby creating and extending partnerships with volunteers have become increasingly urgent [17]. Participation of volunteers in flood forecasting exists in Central America, especially in Honduras, but the scope of using them in a FEW dissemination process has not been addressed in detail [2]. In a study on Community-Based Flood Early Warning Systems (CBFEWSs), the CBDM set-up in Nepal was found to be an effective mechanism in flood warning dissemination and response [18]. These CBDM committees comprise local media, the Red Cross, local police, military units, and forecasting stations of the Department of Hydrology and Meteorology. However, there is no involvement of the local resource of organized and unorganized volunteers in these committees. The only voluntary organization engaged in the committee is the International Federation of Red Cross (IFRC). However, the IFRC has a different definition of a CBEWS. For the IFRC, a CBEWS does not imply the participation of the community but merely refers to a system where any other organizations or agencies of the community implement the task on behalf of the community [19]. In the existing rich literature on FFEWS, there are other examples where volunteers contribute to the flood forecasting process, but none claimed the involvement of local volunteers in the FEW dissemination, except the cloud-based FEWS (the latest version of technology based real-time FFEWS, participated in by individuals voluntarily using Google Drive’s analytical features) [20]. However, this does not take place in a social context and unlike other ‘pro-social actions’ needs ‘a planned action’ and therefore cannot be considered as voluntarism [21]. Hence, therefore, a knowledge gap exists with regard to using the local resource of volunteers in the dissemination of a FEW.

Risk communication occurs in a social context, which allows information to pass through the responsible authorities to the intended recipients, not only with the help of technology but also through social interaction [22]. Social networks and social capital such as volunteerism can play a role in bridging the gap where technology fails to reach the maximum population. The principle of participation lies at heart of the CBDM [23]. There are numerous examples of volunteers participating in the diverse activities of government and business organizations [13,24]. The volunteers who are dedicated to disaster management are different from other forms of volunteers by virtue of different circumstances and their specific tasks [25]. This study explored the scope for volunteers in disaster management but in the preparedness phase—to be specific, in FEW dissemination processes [25,26]. However, the current study emphasized the positive aspect of this social phenomenon in an important and potentially disastrous issue such as a flood. Therefore, the current study concentrated on the pre-flood warning and information dissemination process, a topic which has gained less scholarly attention in studies on volunteerism. In this study, the existing role of community volunteers was investigated in the FEW dissemination process, the nature and extent of their relations with local government organizations in terms of top-down flood information flows, and the common perception of and potentials for incorporating volunteers into a community-based FEW dissemination system. To explore these issues, the following research questions (RQs)were investigated and answered:

RQ1. What is the role of volunteers in a FEW dissemination in the community level of flood-prone areas in Bangladesh?

RQ2.What is the relationship between local government institutions (in Bangladesh, Union Councils—UCs) and volunteers in terms of a FEW flow?

RQ3. How can volunteers increase the efficiency of UDMCs to disseminate a FEW in a community based flood management set-up?

## 2. Material and Methods

As this study explored how people in a specific social context make meaning, an inductive logic and qualitative method was employed [27].In the inductive approach, after collecting data, the authors developed themes which were used to identify patterns and theories. These themes or patterns were then tested and compared with the relevant literature.

### 2.1. Semi-Structured Interviews (SSI)

In this study, SSIs were used to collect information from participants who have had experience of floods along with volunteering. The reason for choosing an SSI is that they are flexible, accessible, intelligible, and efficient at disclosing important behavioral characteristics [27]. Moreover, an SSI allows opportunities to probe for further clarification to explore complex issues [28]. Each interview lasted for 20 to 30 min. To make the interviews consistent, an interview schedule was used (Supplementary Schedule S1). To make the interviews spontaneous, the interview schedule was not followed rigidly. The participants were allowed to speak their minds as much as they wished, and in fact were encouraged to do so. However, probing was often necessary to keep them engaged. Occasionally, a simplified alternative word was used to make the participants understand some technical words which were not clear to their native language of Bangla (in which the interviews were held), such as ‘early warnings’, ‘volunteers’, ‘flood information’, ‘disaster management committees’, among others. It was an important decision to make whether to conduct the interviews anonymously or overtly. While collecting data, hiding one’s identity is considered a less important ethical issue, but to some scholars it is a serious breach of ethical conduct [29]. Hence, a delicate combination of both was used here, i.e., a limited amount of information about the interviewer was provided. However, on occasions, the provided information gave a slightly different impression among the participants about the purpose of the research. In such cases, further clarification about the purpose of the study was provided.

### 2.2. Location of the Study and Sampling

**Figure 2:**
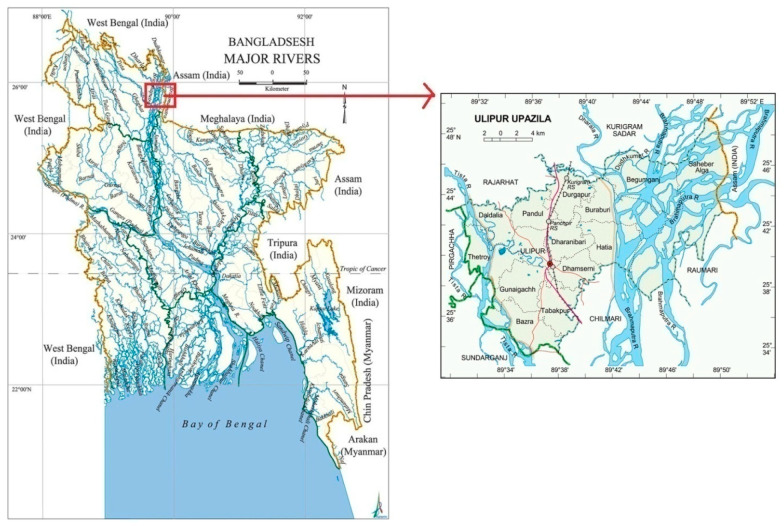
Hydrological system of Bangladesh and the location of the study area.

The data for this study were collected from Ulipur (sub-district) of Kurigram District (Figure 2). Kurigram is a district of Bangladesh that has 18 different sized rivers entering from India and crisscrossing the land mass of all the sub-districts. Ulipur is a sub-district having a population density of 810/km^2^ where floods constantly impact the local people [30]. It has three major rivers: the Brahmaputra, the Dhorola, and the Tista. These three mighty rivers cause floods almost every year to some extent [31]. Moreover, 50% of the area of Ulipur sub-district falls under the ‘Tista Meander Floodplain Agroecological Zone’, and it is known as a ‘flood-prone’ area with an average annual rainfall of about 2931 mm [32]. People here thus have a wealth of experience dealing with the floods and devising coping strategies. All the participants of this study were from different communities of Ulipur.

Considering the target of this study, a purposive sampling method was used to select the participants. Purposive sampling is a non-probability sampling strategy which is also known as judgmental, selective, or subjective sampling. Since proportionality was not the main concern of this study and subjective methods were included to decide the sample, this type of sampling was deemed appropriate [33]. Among the seven types of purposive sampling, expert sampling was used to select the participants because the interview questions may not have been suitable for non-experts. It is usual that many people do not have an interest in disaster management, voluntarism, or local government institutions. Therefore, it was important to identify people with a particular type of knowledge and experience, such as local government institutes (UCs) of volunteerism and floods and who were willing to share it with the authors. The sample size was 10 participants, all residents of Ulipur sub-district. One of them was a government official, a member of the UzDMC, and the chief scout coordinator by designation. He was the first person to respond in the event of any emergency on behalf of the central government in a local government set up. Two participants were chairmen of two different UCs, who were solely responsible for managing administrative and development related activities in their jurisdiction. They were politically related to the wider population and elected directly by them. The next two participants were journalists who were well connected with the local government institutions and to the local populace. They also had experience in volunteering during emergencies alongside the administration. A further two participants were officially involved with a local level volunteer organization called Bangladesh Scouts. They were actively associated with the UzDMC but not with the UDMC and play an important role in post-disaster relief and rescue operations. The remaining three participants were local people with different occupations. They are vocal and widely seen as unofficial spokespeople in their communities. All the participants were permanent residents of different UC areas except the government officer. Since the nine participants were locals and had assets and permanent houses in this sub-district, they experienced varied levels of loss caused by floods over the years. All the participants willingly took part in this study. Before participating in the interview, the respondents were requested to acknowledge their consent verbally according to the consent form (Supplementary Schedule S2). They were also given opportunities to ask any relevant question regarding the purpose of the study. As this study deals only with the behavioral and perceptional issues of individuals, it did not pose any threat to the participants. To keep the recorded interview data secure, the audio files were encrypted, and only the authors had access to them.

### 2.3. Transcription

The recorded data for each interview were translated into English and transcribed immediately so that no comments were omitted and the meaning remained as intended. While the transcribing was undertaken with ‘an appropriate level of detail’, to reinforce the accuracy, the transcripts were checked several times [31]. The answers were organized according to each question asked to the participants. While transcribing, the identity (name and location) of the participant was not mentioned; instead, numbers (1 to 10) were used for the participants. All information that could reveal the identification of the participant was omitted from the interview files. However, some information about the participants’ occupations was left, as it contributed to analyzing the data.

### 2.4. Data Analysis

**Figure 3:**
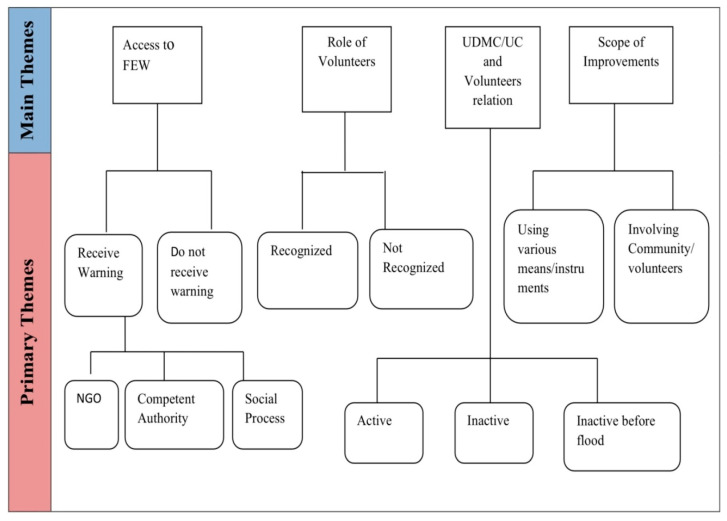
Schematic representation of the thematic map followed in this study to investigate the relationship of community volunteers with FEW.

To analyze the interview data, a thematic analytical approach was adopted. Importantly, while identifying the themes a semantic approach was adopted, meaning that the themes were identified according to the surface meaning of what the participants stated in their statements. The analysis of the data did not look for any ‘inner meaning’ or latent information embedded within the whole data set. In such an analytical process, the collected data is generally organized to describe patterns by describing, summarizing, and interpreting [34]. All transcribed data were uploaded in ‘NVivo’ software, and then the codes were marked within twelve primary themes, which were then gathered into four explicit themes, or main themes, until they represented meaningful patterns (Figure 3). This process followed the guidelines set out by Braun and Clarke [34]. Four themes were identified representing the underlying ideas of the participants. These themes described the present state of the local FEW dissemination processes, the role of volunteers in this process, the relation of the UCDMCs and the volunteers, and the scope for improvement in the FEW dissemination processes (Figure 3). It was noticeable that being isolated from the context of the interview, these themes were no more related to the individuals’ overall statement as a whole.

## 3. Results

### 3.1. Access to FEW

Before discussing the contribution of volunteers in the flood warning dissemination process, it is important to ascertain whether people have access to flood warnings or not. People expect early warnings before any natural disaster. Therefore, the government and other agencies responsible for disaster management emphasize forecasting and warning. However, most of this study’s participants did not acknowledge a FEW from any official sources. When asked how they receive flood warnings, one participant (No. 1) who had received a FEW replied as follows:

We receive information of the water levels of the major rivers on a daily basis but during the rainy season we get hourly updates. Some of the information comes from the concerned ministries and the hydrological authority, some from the local leaders and some from locals living near the river banks. They send me information by email and fax, sometime I get phone calls. As I am directly related to the disaster management set up of this area, people keep sending me updates.

However, a majority of the participants said that they did not receive any FEWs. This theme had a number of additional findings. Two of the participants who agreed that they received some sort of FEW noted that this information came from informal sources such as relatives, local political leaders, and people they met in their leisure time. One participant (No. 4) said,

We receive flood related information from various sources, for example local politicians or people living close to the rivers and remote villages. They are the first to inform us about the water level rise and estimated damage. But we do not get any information directly from the Upazila Disaster Management Committee or Union Councils. I have heard of some mobile messages but I think everyone do not get messages, never heard of people talking about receiving such messages.

One of these two participants (No. 8), who worked in farming and was well connected with local officials responsible for agriculture, said he received early warnings from a different kind of office unrelated to the state’s flood forecasting and warning mechanism. Participants who did not get a FEW added that they use ‘local knowledge’ to predict the severity of a flood, although this knowledge is often not enough to give them sufficient time to save their crops.

Channeling an official FEW is a top-down information flow in Bangladesh. The concerned ministries and the hydrological authorities provide information to be broadcasted on TV and radio channels. However, a great number of participants said that they did not receive specific warnings through these channels. To some participants (Nos. 5, 8, and 9), this information was merely bad news, not a FEW. When asked about the function of TV, radio, and newspapers in disseminating a FEW, one of the participants (No. 10) stated the following:

The news we get from TV and radio channels is not useful. What use is a flood early warning when it is already going on? People need information at least 5 to 7 days earlier so they can save their assets and crops.

In addition, these FEWs are not specific to any particular area; they are mainly general information about the severity and losses caused by the flood. However, access to TV and newspapers was an important point. As one participant (No. 8), a farmer, said:

I mainly get the overall flood situation of the country from TV but the agricultural extension office provides me with important instructions that are helpful for my farm. There is a problem with the TV; we do not get continuous electricity. Whenever there is a strong wind or rain starts, the TV signal is interrupted.

When asked, one participant (No. 4) had a different opinion of the effectiveness of TV:

…TV and radio news are enough to make people warned about the upcoming flood, but people do not realize the importance of these messages. That is why most of the time people are not adequately prepared.

Half of the participants considered TV bulletins less useful. One participant (No. 5) said the following:

From TV and radio we come to know mostly about the areas where the flood has already hit. Sometimes there are programs where they tell us how to prepare before a flood, but we don’t see them coming very often. Most of the time, we miss them because we remain busy in our daily work.

### 3.2. Role of Volunteers

**Figure 4:**
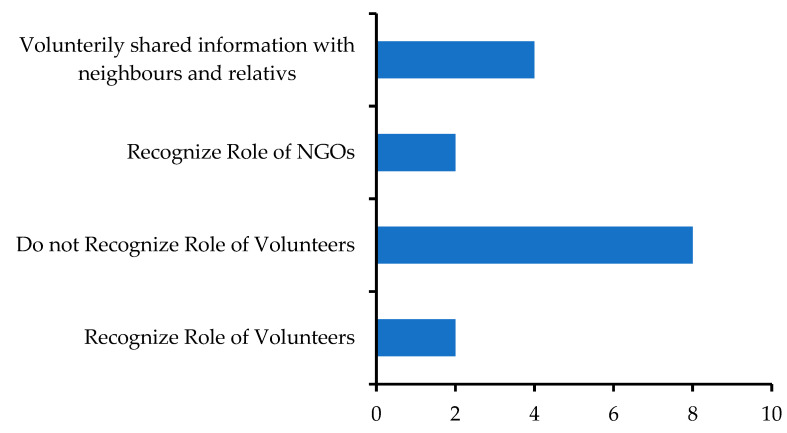
Recognizing the role of volunteers in FEW. Responses from the participants of this study.

This theme directly answered the RQ1. While most of the participants were of the opinion that volunteers were not involved in the FEW dissemination processes, a number of them acknowledged that they had shared flood-related information with their relatives and in the community when they came to know about an impending flood (Figure 4). The two participants (Nos. 6 and 7) who were members of Bangladesh Scouts did not recognize any role for volunteers in disseminating a FEW. One of them said,

There is no specific volunteer group in our area working independently in a disaster situation. But we see NGOs who play a benevolent role before, during and after a flood. They warn people about the destruction of floods, arrange health training and provide relief to the poorest. They also take part in the rehabilitation process. We see Bangladesh Scouts and Red Cross taking part in the relief distribution. But none of them have any mentionable activities prior to a flood event.

In contrast, two participants (Nos. 1 and 3) recognized the role of volunteers in FEW dissemination. One of the participants (a member of a UDMC) noted,

There are some volunteers who work to create awareness among school children. These volunteers are driven by some teachers who are involved with voluntary organizations. These types of activities are highly encouraging, but it is not a regular phenomenon.

However, further probing disclosed that when the participants (Nos. 8 and 9) came to know about heavy rain or flash floods in India, they started warning their family members and relatives, as there was a great possibility of that flood water hitting their lands. Yet they do not get such information very often.

As noted in Section 2.1, all the participants had some kind of experience in volunteering in their locality and were well-aware of their importance in emergency situations. They (Nos. 3,4,5,7, and 10) actively took part in post-flood situations. Still, they were hardly aware of disseminating FEWs and their role in it. When asked, one of the participants replied,

I didn’t realize it earlier. But I am not authorized to disseminate any flood-related warning to the people.

Another participant (No. 7) said,

I can help if the government wants me to.

However, all the participants were of the opinion that they could play role in disseminating FEW if they were properly instructed from a competent government agency. While describing the role of volunteers, one participant (No. 1) mentioned that FEWs were sent to the UCs to take precautionary measures. The UCs held meetings and took the necessary action to disseminate FEWs. He said,

We (UzDMC) pass required information and instructions to the UDMCs for wide circulation among the populace. These committees take initiatives to pass the information to the masses using microphones and other means… It is not well organized but UCs are solely responsible for that.

From this theme, public perception of the role of volunteers in a FEW dissemination can be identified. There are important findings about volunteers. First, there is no active role of volunteers in flood warning dissemination. Second, people spontaneously disseminate information that they have among their family members, relatives, and neighbors. Third, uncoordinated, some NGOs play a role in the flood information dissemination process. Fourth, the traditional top-down FEW system does not reach the mass of the population and does not use local people or volunteers. Five, people are willing to take part in the dissemination process, but they require a valid platform for doing so.

### 3.3. Relationship between the UDMC and Volunteers

To address RQ2, the participants were asked to state their understanding of the role of the UDMC prior to any flood event, the participation of local people, and how volunteers were connected with the UDMCs.

Seven of the participants did not recognize any action of the UDMCs before a flood event, but in contrast (and perhaps predictably), participants (Nos. 2 and 3) who were members of the UDMCs claimed the UDMCs were active before a flood event. One of them said the following:

We have standing orders for UDMCs. There are clear instructions to arrange meetings in the UCs and plan for an upcoming disaster event. It is our responsibility to disseminate flood warnings among the populace although we don’t know about any prescribed method for doing it. Within our limitations, when we get a FEW we try to disseminate the information using microphones and sending information to the remotest areas. Not every committee does the same. It depends on the decision of the committees.

However, other participants gave quite the opposite reaction:

UDMCs are not very active. They are supposed to sit in a meeting every month but as far I know they only arrange meetings in emergencies. Most of the time, the main agenda is to formulate plans for relief distribution or recovery actions.

Another central concern of this study was to reveal the relation of the UDMC with the masses as well as the volunteers from the communities. In addition to the three participants who were member of the UzDMC, seven other participants claimed to have no direct involvement with the UDMCs:

I do not have any direct contact with the UDMCs but I am informed about their activities very often. I communicate with the committee members to get updated information of the overall flood situation and the role of the government. I am not a part of the committee. But they ask for my help in the relief and rehabilitation process. I like to help the community people.

This participant claimed that the UDMCs have not yet developed any mechanisms to incorporate the pool of volunteers in any activities other than relief distribution and the subsequent rehabilitation process.

However, voluntary organizations such as Bangladesh Scouts and the IFRC take part in the UzDMC meetings. Different NGOs are also a part of this committee and their role is also limited to relief distribution and rehabilitation:

Various NGOs and voluntary organization such as Bangladesh Scouts and the IFRC attend the UzDMC. They share their information and receive instructions. They usually prepare for relief and rescue operations but at the community level the participation of the volunteers is spontaneous. They are not organized; they just appear after the disaster and take part in the relief and rescues missions.

This theme had several findings related to RQ 2. First, the UDMCs were not sufficiently active before an impending flood. Second, community participation could help the UDMCs to be more active in preparing for a flood event. Third, community volunteers had no links with the UDMCs. Fourth, although NGOs and other formal volunteer groups were involved with the UzDMC, their function was limited to the post-flood response phase.

### 3.4. Scope for Improvement

This theme addresses RQ3. It seems that all the participants realized the importance of an effective FEW system and had a positive view of it. In order to make the UDMCs more effective at disseminating an effective and holistic FEW, the participants expressed that the UDMCs have significant scope for improvement. One said,

UDMCs are the last stage of the top-down disaster management process of the government. The success of flood management largely depends on these institutions. People reap benefits from the actions of these committees. That is why the accountability of these committees is an important factor. But they are hardly aware of the pre-flood measures that can decrease losses. Volunteers would be happy to get involved in such activities.

Not only increasing accountability and widening scope, participants also thought that the weakness of UDMCs could be reduced if community people would get a proper chance to contribute alongside the elected members of the union council:

The UDMCs are weak at performing their duties. Though they have clear instructions, they hardly follow them. Very few people know about their activities. If other NGOs and local individuals can be involved in this committee, I think they can perform better.

When asked about the potential ways of disseminating a FEW to the maximum population, the participants agreed that mobile messages could be the easiest way. However, they also believed that this method would not reach the poorest and most uneducated sections of the community. Moreover, although many people use mobile phones today, there are still occasions when text messages may be missed. In addition, SMSs are too short to provide the requisite amount of detailed information. Furthermore, it is a one-way approach; people cannot get answers if they have any queries. One participant (No. 7) said,

I think mobile messages can reach me quickly, but I may miss it. We get lots of SMSs daily. We do not pay too much attention to them. So, it is better to make arrangements for public announcements using microphones. In this way there will be little chance to miss the information.

**Figure 5:**
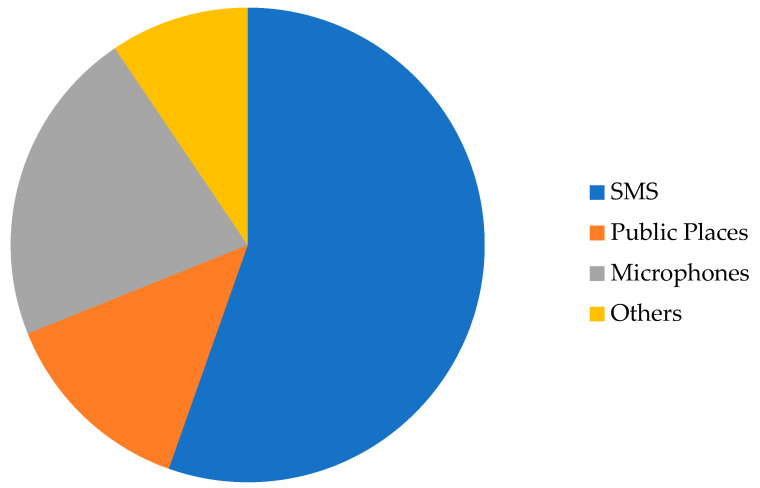
Expected means of communicating a FEW. Responses from the participants of this study.

Five of the participants emphasized the use of microphones in addition to SMS, and two mentioned local leaders and members of the UCs, the village police, and NGOs, while others held different opinions (Figure 5).

Although initially none of the participants spoke about any kind of social processes, they did begin to speak positively about employing volunteers and NGOs when probed. They also recognized the role of religious leaders (Imams) and teachers. Some suggested a ‘door-to-door’ approach:

…volunteers are good people. They are neutral and people have faith in them. They can go door-to-door because they know well who needs to be warned. If they use the microphones of the mosques and the UCs, it will cover maximum people; and people can also ask them questions if they have any.

However, few participants were not without doubts about the authority of volunteers to do this efficiently. They even thought that the existing nature of volunteerism was not capable of performing such actions:

…they can be good help but they are not well organized in our rural community and you cannot always get them around. We can make separate committees in our villages and train them. But who would believe them if they are not empowered by any competent authority?

In contrast, the members of both disaster management committees (UzDMC and UDMC) believed that volunteers have always been very helpful in implementing various government agendas and they could easily be organized in a single platform to assist the UCs in managing disasters.

In this work, four main themes were identified to answer the research questions. The first theme was not directly related to any of the three research questions, but it provided important information about the overall study. The second theme found that community volunteers did not take part in any pre flood preparedness activities, but they participated in the post-disaster response phase. The third theme identified that the UDMCs were not active enough, and that one of the reasons for this was that they did not involve community participation or volunteers in the pre-flood preparedness phase. The fourth theme suggested scope for how the FEW dissemination systems could be improved.

## 4. Discussion

In the preparedness phase of a flood, to be specific, in FEW dissemination processes, scant scholarly attention to the role of volunteers has been paid. Therefore, this study explored the social context of the community of a flood-prone area of Bangladesh and investigated the current state of its FEW dissemination system, the role of the volunteers in this processes, the relationship between the CBDM institutions and volunteers, and the scope for improvements that may be made by integrating local volunteers into the system. Connecting to the research questions, four themes were identified (Section 2.4; Figure 3), which are discussed below.

### 4.1. Access to a FEW

For an agro-based economy like Bangladesh, FEW is an important issue, as it increases the safety of millions of people and reduces damage to assets [3]. Therefore, access to FEWs is vital for people living in flood-prone areas. In this study, it was found that people living in a flood-prone community may not receive timely and effective FEWs through the traditional and technology based top-down channels. This finding is similar to the study performed by Fakhruddin and Ballio [6], where they studied the capacity and needs assessment of five flood-prone communities (sub-districts) in Bangladesh. In addition, this study found effective the roles of local communities and NGOs, local police forces, and agricultural extension offices in the flood information dissemination processes, which also corresponds well with the outcomes reported by Fakhruddin and Ballio [6]. In local communities, locally managed and circulated flood information is preferred since they encounter issues accessing the technology-based means of FEWs [9]; therefore, it reassures the vital task to institutionalize community participation in the UCs to make UDMCs more efficient. Since community participation is a key resource in DRR [6], this study identified and explored sound grounds for involving community volunteers with UDMCs for progressive improvement of disseminating FEWs.

### 4.2. Role of Volunteers

The exploration of the role of volunteers in disseminating a FEW to answer RQ1 did not find a prominent role of volunteers prior to a flood event but did find this social behavior to be evident but limited to neighbors and relatives. In some cases, the voluntary contribution of teachers and local politicians was acknowledged, as was the contribution of NGOs. However, none of these actors are active enough to be formally defined as engaged in voluntary activity. This type of community-led flood warning dissemination was already acknowledged previously as an ‘informal warning’ [35]. However, this kind of informal warning process can raise problems such as authenticity issues, lack of information, and the possibility that false alarms are generated [9]. Moreover, communities lack sufficient technical knowledge to understand or update flood forecasting. Therefore, it is essential to combine these informal sources of flood warning with official channels [36]. The participants in this study fully realized the necessity of organizing community volunteers. The voluntary attitude of the local people can be amplified and reinforced by being institutionalized under the umbrella of UCs, which is hence recommended.

### 4.3. UDMC and Volunteer Relations

This study considered UCs as the spokespeople of the community. They are responsible for local disaster management through UDMCs. This investigation found that in the post-flood response and recovery phase, UDMCs are suddenly rendered very active supports, and volunteers work with and through them. Bangladesh Scouts and the IFRC actively take part in UzDMC meetings and engage themselves to offer help to the community although they do not collaborate with UDMCs, which are the center of the communities and the basis of Bangladesh’s CBDM approach. From the participants’ responses it was evident that although there is enough scope to include local people into UDMCs, as yet, this is not the case. However, it was found that UDMCs are too weak to support communities in a pre-flood situation. One of the reasons for this is that this macro-level local government body is not included in the list of organizations that receives flood bulletins from the FFWC of Bangladesh [2]. In the FEW dissemination model of Bangladesh, the UCs are related with local branch offices of NGOs, and NGOs take part in the dissemination process through their field employees and volunteers [7]. Furthermore, it needs to be ensured that the UCs have an appropriate and effective channel to receive FEWs through top-down approaches.

### 4.4. Scope for Improvement

This study found sufficient scope for improvement in the dissemination of flood warning messages. First, the inactive state of UDMCs needs to be addressed before an impending flood, allowing it to work as a common platform interlinking all social and administrative structures and organizations. Second, UDMCs need to create more scope for involving volunteers to formulate FEW messages and plan for and be intimately involved in their dissemination. Finally, UDMCs need to use various instruments considering the needs of the community. This study’s participants recommended that volunteers use microphones and go door-to-door in at-risk areas to warn people. They also suggested that warning messages be delivered in public spaces such as markets or mosques; however, such improvements were also recommended previously.

The strengths of this study are two-fold: the identification of the geographical area; and the selection of the participants. Ulipur, as a study area, best represents the flood-prone areas of Bangladesh and has a typical CBDRR mechanism that resembles other areas nationwide. The study’s participants were also carefully chosen, including both flood victims as well as potential volunteers in an emergency situation who are also connected with community disaster management processes. The study’s limitations are no less important. Firstly, if the number of the participants were larger, the results would have been more pragmatic. Secondly, it would have been more informative if this study had included participants who work in the policy formulation process of the central administration and disaster management board.

## 5. Conclusions

Though the role of volunteers is a nuanced topic, it has received less importance in the preparedness phase of disaster management, especially in terms of a FEW dissemination. Considering this, the current study focused on the perception of incorporating volunteers prior to an impending flood event and revealed that the perceptions of the would-be volunteers are very strong and positive in connection to the flood risk communication. Further, it was also observed that active inclusion of volunteers in the UDMCs needs to be practiced in the form of a dedicated national disaster volunteer group, which is already prescribed in the national policy document, however yet to be implemented. Nevertheless, it would be imperative to train volunteers of flood-prone communities for enhanced flood risk communication. Moreover, further research is needed in the dissemination endeavor. Finally, this study verified the necessity of granting a legal door to volunteers to establish a dedicated national disaster volunteer group effective for all sorts of emergencies.

## Data Availability

All processed data were presented and described in Section 3 (Results). However, the data that support the findings of this study are not publicly available because we did not ask participants to consent to raw data sharing outside of the research team. Public sharing of the data could compromise anonymity and research participant consent.

## Supplementary Materials

The following are available online at https://www.mdpi.com/article/10.3390/ijerph182413010/s1.
