# Supplementary material for: Potential of Community Volunteers in Flood Early Warning Dissemination: A Case Study of Bangladesh
Source: Int J Environ Res Public Health. 2021 Dec 9;18(24):13010. doi: 10.3390/ijerph182413010 (PMC8700901; doi:10.3390/ijerph182413010)
Supplement: Supplementary file 1 [file ijerph-18-13010-s001.zip › ijerph-1470045-supplementary.pdf]

Manuscript: IJERPH- 1470045

Title: Potential of Community Volunteers in Flood Early Warning Dissemination: A Case Study of Bangladesh

Supplementary schedule S1

Interview schedule

1. How do you get flood early warnings?
2. What types of information do you get from TV, radio or SMS about the impending flood?
3. How do volunteers play role in disseminating flood early warnings?
4. How can you contribute to disseminate flood early warnings?
5. What type of involvement do you have with the union disaster management committee?
6. What is the role of union disaster management committee prior to a flood event?
7. How do you think volunteers are associated with the union councils?
8. What are the scopes of improvement in the union disaster management committee?
9. How do you think flood early warning messages can be sent to you easily?
10. How community volunteers can be involved in disseminating flood early warning messages?
11. What can be done to ensure participation of the volunteers in flood early warning dissemination process?
12. What means can be used by the volunteers to reach maximum people at flood risk?

Supplementary schedule S2

Consent Form

If you are happy to participate please complete and sign the consent form below

	**Activities**	Initials
1	I confirm that I have read the attached information sheet for the above study and have had the opportunity to consider the information and ask questions and had these answered satisfactorily.	
2	I understand that my participation in the study is voluntary and that I am free to withdraw at any time without giving a reason and without detriment to myself. I understand that it will not be possible to remove my data from the project once it has been anonymised and forms part of the data set.<br><br>I agree to take part on this basis.	
3	I agree to the **interviews** being **recorded**.	
4	I agree that **pictures of my face** may be used in publications.	
5	I agree that any data collected may be published in anonymous form in **academic books, reports or journals.**	
6	I understand that data collected during the study may be looked at by individuals from The University of Manchester or regulatory authorities, where it is relevant to my taking part in this research. I give permission for these individuals to have access to my data.	
7	I agree that any **personal/anonymised** data collected may be shared with **researchers/researchers at other institutions**.	
8	I agree that the **researchers/researchers at other institutions** may contact me in future about other research projects.	
9	I agree that the researchers may retain my contact details in order to provide me with a summary of the findings for this study.	
10	I understand that there may be instances where during the course of the **interview/focus group**informationis revealed which means that the researchers will be obliged to break confidentiality and this has been explained in more detail in the information sheet.	
11	I agree to take part in this study.	

Data Protection

The personal information we collect and use to conduct this research will be processed in accordance with data protection law as explained in the Participant Information Sheet and the Privacy Notice for Research Participants.

____________________ ____________________ ____________

Name of Participant Signature Date

____________________ ____________________ ____________

Name of the person taking consent Signature Date

[Insert details of what will happen to the copies of consent form e.g. 1 copy for the participant, 1 copy for the research team (original), 1 copy for the medical notes]
